# A 13-year patient journey of infant giant clival chordoma: case report and literature review

**DOI:** 10.1007/s00381-022-05749-4

**Published:** 2022-11-22

**Authors:** J. Apps, R. Gagen, E. Neumann, G. Solanki, M. English

**Affiliations:** 1 Department of Oncology, Birmingham Women’s and Children’s NHS Foundation Trust, Birmingham, UK; 2 Department of Neurosurgery, Birmingham Women’s and Children’s NHS Foundation Trust, Birmingham, UK; 3 Radiology Department, Birmingham Women’s and Children’s NHS Foundation Trust, Birmingham, UK; 4 Cancer Research Clinical Trials Unit, University of Birmingham, Birmingham, UK

**Keywords:** Infant clival chordoma, Imatinib, mTOR

## Abstract

Chordomas are rare malignant bone tumours that develop from the ectopic remnants of the embryonic notochord. In contrast to adults, the majority in children under 16 present intra-cranially (63%). In 2006, we reported the youngest case of a large clival chordoma, a 15-week old baby, the second case to present without skull base involvement and the fourth case of chordoma in a patient with tuberous sclerosis (TS) Kombogiorgas (Childs Nerv Syst 22(10):1369–1374, 2006). In this report, we provide an update on this patient’s journey through a range of therapeutic options and summarize an update of the literature, since 2006, for this patient group.

**Supplementary Information:**

The online version contains supplementary material available at 10.1007/s00381-022-05749-4.

## Case report

**Fig. 1:**
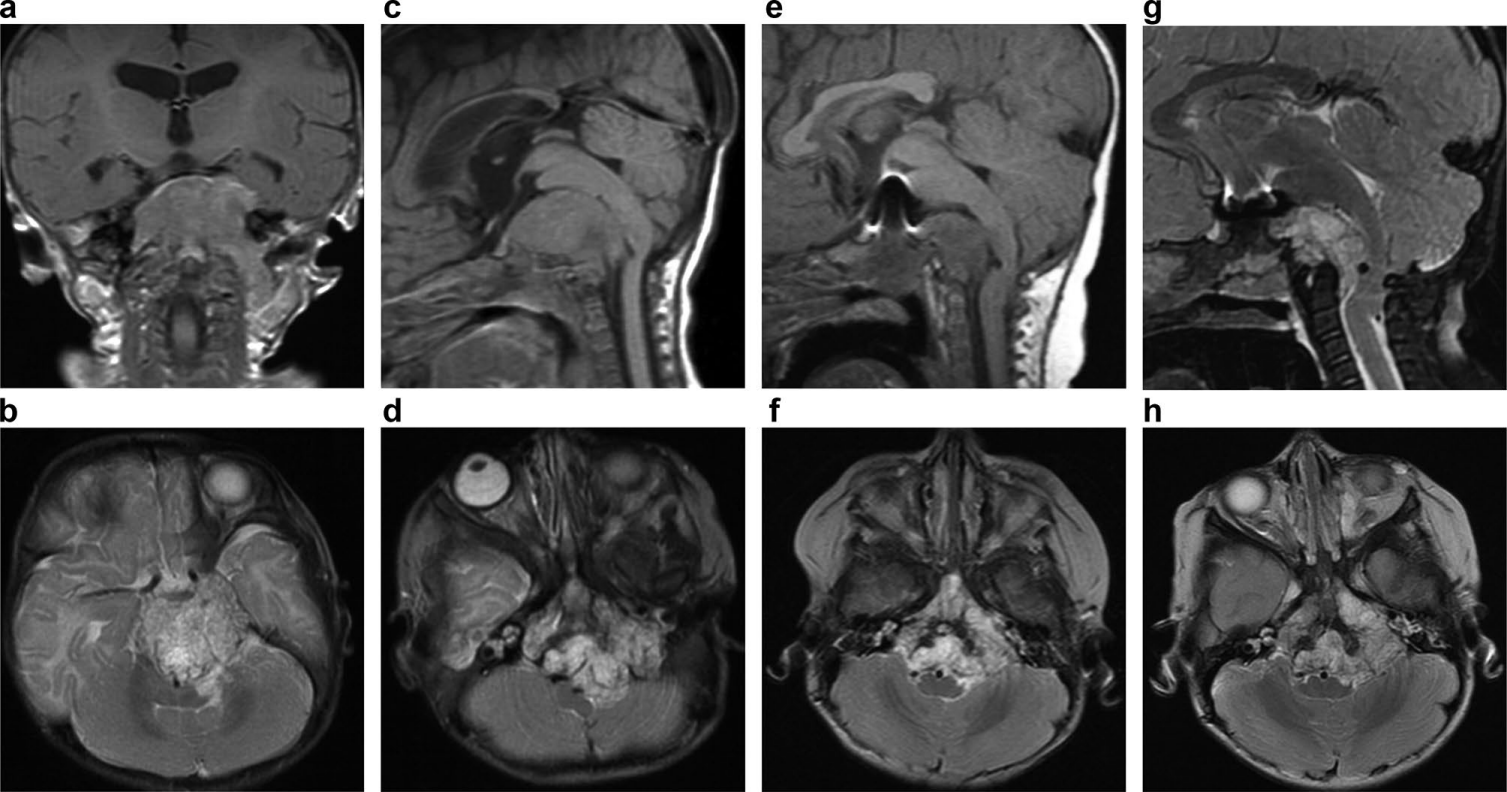
**a**–**d** Initial diagnostic imaging demonstrating a heterogenous mass centred on the clivus: **a** Coronal T1 post-contrast imaging showing homogenous lesion enhancement and extension of the mass through the left jugular foramina. **b** Axial T2 weighted image; the mass extends anteriorly towards the left cavernous sinus. **c** Mid-sagittal T1 weighted image, and **b** axial T2 weighted image demonstrating compression of the brainstem. **e**–**f** Reduction in size and associated mass effect following initial treatment. **e** Mid-sagittal T1 weighted image; metallic artifact secondary to a surgical clip is demonstrated antero-superior to the lesion. **f** Axial T2 weighted image. **g**–**h** Reassessment imaging following completion of carboplatin/etoposide treatment showing stable disease. **g** Mid-sagittal T2 fat-sat image. **h** Axial T2 weighted image

This boy presented at 15 weeks of age with an 8-week history of persistent cough, difficulty in feeding, and faltering growth. MRI demonstrated a lobulated heterogeneous mass contiguous with the clivus displacing the midbrain and brain stem posteriorly with hydrocephalus and possible intracranial and spinal metastases (Fig. 1a, b, c, and d). He underwent a third ventriculostomy and an endoscopic biopsy of the tumour, which confirmed a diagnosis of chordoma. He was diagnosed with TS, based on a strong family history, and the presence of cardiac rhabdomyomas and subependymal nodules [1].

Initial management was with ifosfamide and doxorubicin, along with intrathecal methotrexate, hydrocortisone, and cytarabine (for doses, see Supplementary Material). There was some reduction in tumour size, and after 12 cycles (1 year) of treatment, the tumour remained stable (Fig. 1e, f). In view of concerns regarding further toxicity with ifosfamide and doxorubicin, he received a further 8 cycles of 3–4 weekly cycles of carboplatin and etoposide, based on experience in Toronto (Eric Bouffet, personal communication). There was a possible slight reduction in size (Fig. 1g, h); however, the tumour was not amenable to surgical resection, and after discussion, he was not felt suitable for radiotherapy.

**Fig. 2:**
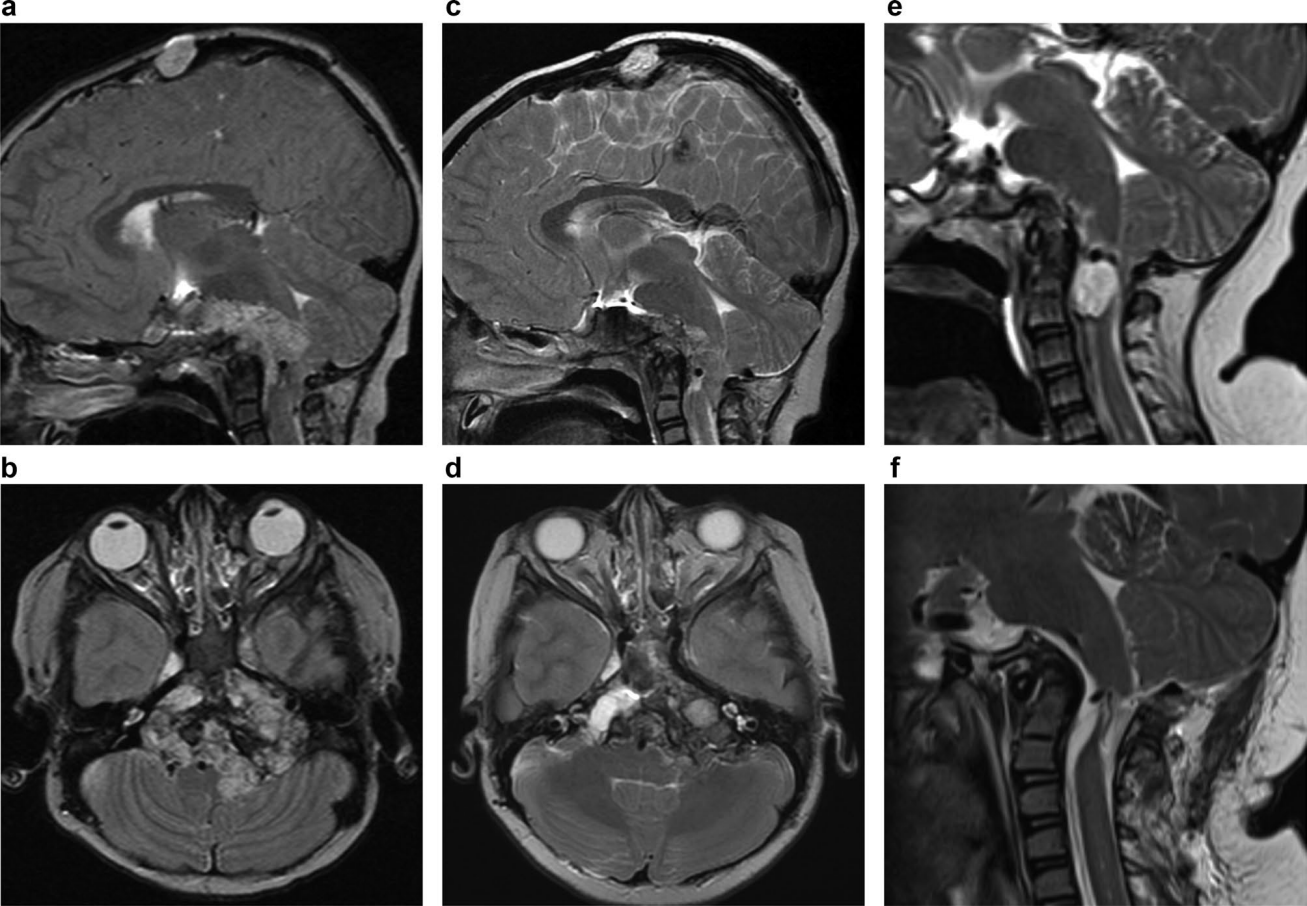
**a**–**b** Primary tumour progression aged 5 years: **a** mid-sagittal T2 weighted imaging showing increased size of the primary mass and a metastatic lesion of the skull towards the vertex. **b** Axial T2 weighted image. **c**–**d** Imaging after 5 months of imatinib and sirolimus showing some minimal reduction in size. **c** Mid-sagittal T2 image. **d** Axial T2 weighted image. **e** Mid-sagittal T2 weighted imaging at 10 years of age showing progression of a nodule to the left of the midline at C1/2 with associated compression of the cervicomedullary junction. **f** Post-operative mid-sagittal T2 weighted image showing nodule resection

His tumour then remained stable until the age of 5 years and 2 months, during which he grew and developed. MRI showed a slight increase in the size of his primary tumour but also three small nodules either involving or just deep to the skull (Fig. 2a, b). He underwent an attempted biopsy, but intraoperatively it was felt not safe to proceed, due to close relations with the sagittal sinus. Further imaging revealed growth of these lesions over the next 6 months, and he was initiated on imatinib and sirolimus, based on his underlying diagnosis of TS and the limited evidence of the use of PDGFR inhibitors and mTOR inhibitors in chordoma [2, 3]. Imaging after 5 months of treatment showed interval improvement in the intracranial appearances with the clival-based tumour enhancing less and being of reduced bulk, reduction in enhancement and reduction in the size of some of the skull vault lesions, and neurological improvement with limited independent walking (Fig. 2c, d). The limited response continued on subsequent imaging. Imatinib was empirically stopped after 1 year, and sirolimus continued. Six months later, there was a slight progression of the clival lesion, and following an episode of optic neuritis, which was attributed to sirolimus, he was switched to a single agent imatinib. He clinically improved, and scans were stable for the next 18 months, following which there was a slight increase in a nodule at the base of the skull at the foramen magnum which slightly compressed the brain stem. This continued to increase over the next 6 months following which he changed to everolimus.

**Fig. 3:**
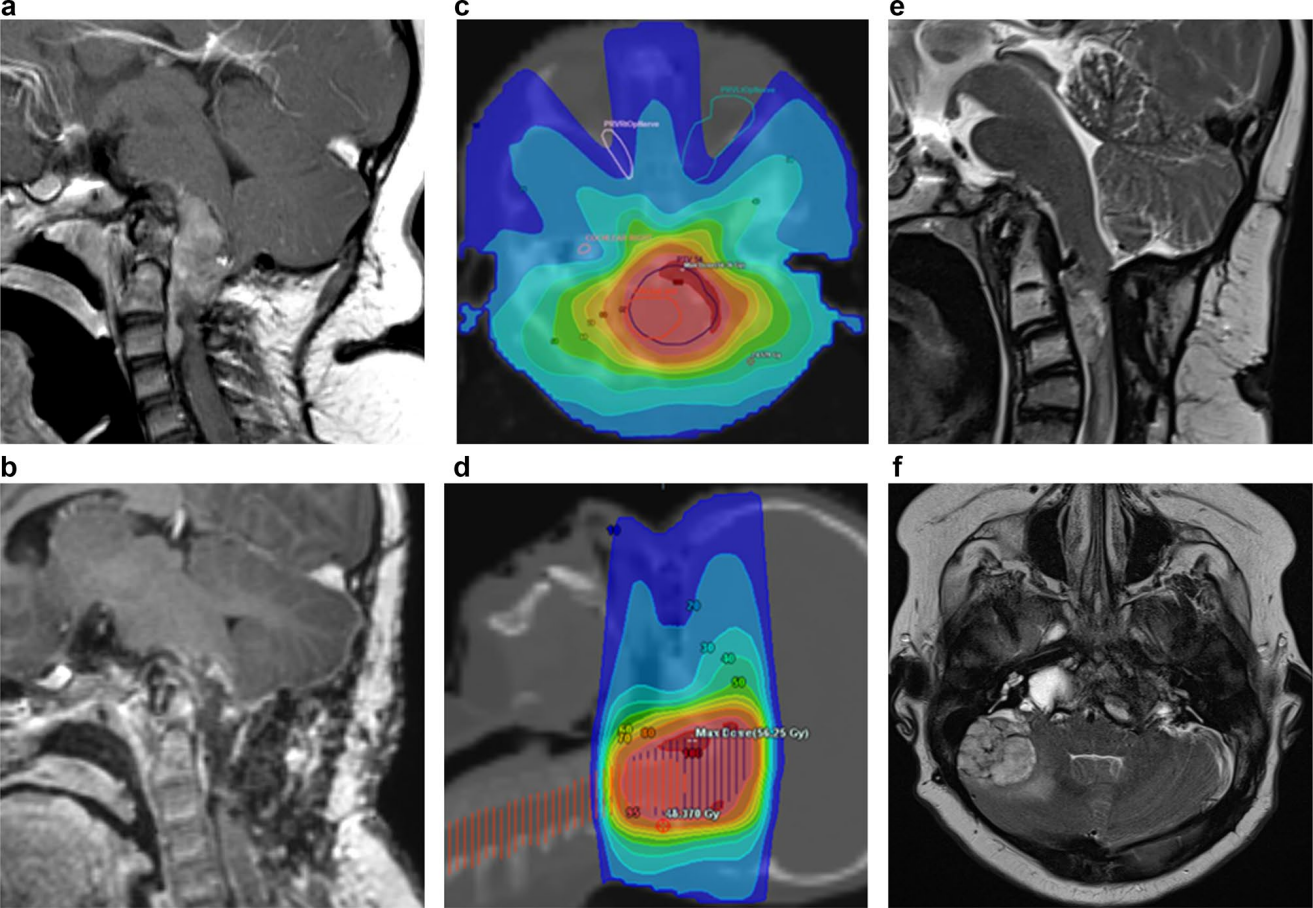
**a** Post-contrast sagittal T1 image showing enhancing recurrent disease at 12 years of age. **b** Post contrast sagittal T1 image showing the results of tumour debulking. **b**–**c** Fused CT/radiotherapy planning maps. **b** axial and **c** sagittal images. **d**–**e** Recurrent disease at 13 years of age: **d** mid-sagittal T2 weighted image demonstrating new cervical disease at C2/3. **e** Axial T2 weighted image showing extra-axial disease overlying the right cerebellar

One year later, following a slow increase in size and concern about increasing compression of the brain stem and spinal cord and increasing neurological signs, he underwent a complete surgical resection of the lesion at the foramen magnum just prior to his 11th birthday (Fig. 2e, f). Unfortunately, there was progressive regrowth of this nodule (Fig. 3a). He underwent a further resection just over 1 year later and received 54 Gy photon radiotherapy (Fig. 3b, c, and d).

Sadly, following radiotherapy, there was further progression in a region anterior and to the right of the cervical cord to the level of the odontoid peg (Fig. 3e). A new area of tumour indenting the right cerebellar hemisphere laterally also developed (Fig. 3f). Over the next 2 months, he developed a right hemiparesis and was initiated on dexamethasone and oral etoposide; however, 1 week later, he developed significant breathing difficulties, likely due to compression of the tumour at the foramen magnum, and upper cervical cord and died aged 13 years and 3 months.

Subsequent to the initial publication of this case, there have been further case reports of chordoma in association with TS, with a total of 13 cases identified [4]. A study comparing these cases with 65 others within in the SEER database highlighted a younger age atpresentation (median 6.2 months vs 12.5 years and more common at the sacral site (40% vs 9.4%) [5].

Maximal resection followed by high dose radiotherapy (70–74 Gy) is the first line therapy for patients with chordoma, though complete (R0) resection is rarely achievable in intracranial cases, and as highlighted by this case, young age may limit the ability to deliver radiotherapy [6, 8]. Chordoma is a chemo-resistant tumour and the majority of evidence relating to chemotherapy is from case reports with only one phase II study, where only 1 response to nitrocamptothecin out of 15 patients was reported [9]. Management of this case was therefore based on limited case reports and expert international opinion. Subsequently, there have been further paediatric case reports and case series of a response to chemotherapy, including ifosfamide, doxorubicin, carboplatin, and etoposide-containing regimens [6, 10–12].

**Fig. 4:**
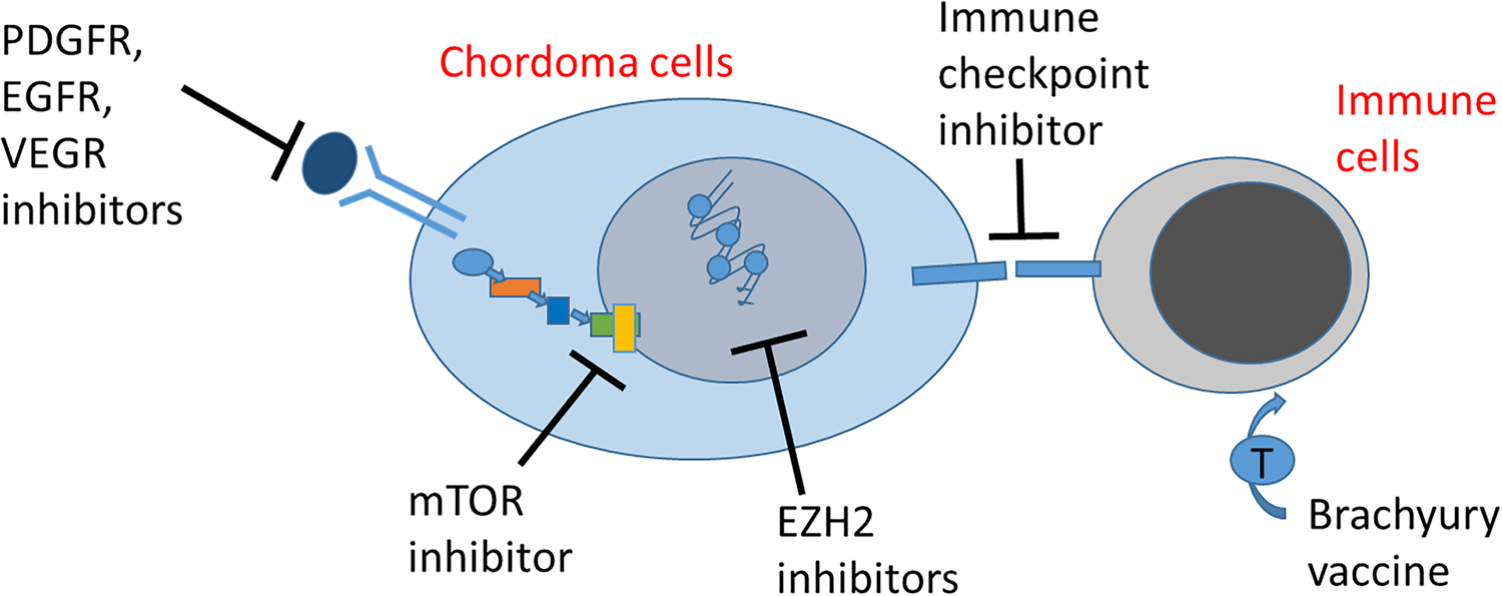
Novel therapies are currently under development for chordhoma. In addition to targeting PDGFR and mTOR pathways, a range of other targets have and are being evaluated, including EGFR, angiogenesis, EZH2 inhibitors, immune checkpoints, and bracyhury (T) vaccine. Further details can be found in Frezza et al. [14] and Hoffman et al. [7]

Since the presentation of this case, there have been a large number of publications and several clinical trials investigating a range of molecularly targeted agents; however, to date, there are no approved agents for chordoma, and their efficacy has been limited [7, 13–15]. Imatinib has been used in over 200 patients; however, response rates were low, though possibly with some association with PDGFRβ expression [7, 13]. Consensus guidelines for the management of relapse have agreed that imatinib and sorafenib are reasonable palliative treatment options [16]. Experience in paediatric patients remains limited, though there are increasing numbers of case reports of the use of imatinib [6, 12, 17, 18]. Whilst results of mTOR inhibitors have been disappointing in chordoma [7, 15], one prospective study evaluated the combination of imatinib and everolimus in 43 adults with advanced chordoma and identified a response rate of 20.9% by the Choi criteria with some association of response to activation of the mTOR pathway [19]. The applicability to patients with associated TS is not clear. In addition to targeting PDGFR and mTOR pathways, a range of other therapeutic approaches are being evaluated which are summarised in Fig. 4 [7, 14].

In summary, chordoma remains challenging to treat in children, particularly where surgery cannot achieve complete resection and radiotherapy is not deliverable. This case adds to an increasing literature describing limited responses to chemotherapy and targeted treatments. Further novel treatment approaches are required and are currently under development.

## Supplementary Information

Below is the link to the electronic supplementary material.

Supplementary file1 (DOCX 12 KB)

## Data Availability

Not applicable.
