# Supplementary material for: A 13-year patient journey of infant giant clival chordoma: case report and literature review
Source: Childs Nerv Syst. 2022 Nov 22;39(4):1077–81. doi: 10.1007/s00381-022-05749-4 (PMC10159969; doi:10.1007/s00381-022-05749-4)
Supplement: Supplementary file 1 — Supplementary file1 (DOCX 12 KB) [file 381_2022_5749_MOESM1_ESM.docx]

**Supplementary material:**

Chemotherapy dosing schedules:

Ifosfamide and Doxorubicin: 3-4 weekly cycles of Ifosfamide (1.5 grams/m^2^ X 3) with MESNA with Doxorubicin (15 mg/m^2^ X 2) with Cardioxane (150 mg/m^2^ X 2). Note ifosfamide and doxorubicin dosed at 50% of standard dose as patient between 3 and 6 months of age.

Carboplatin and Etoposide; 3-4 weekly cycles of Carboplatin: AUC 6.6mgs/ml x minutes (approx. 500mg/m2, etoposide (5mg/m^2^ x 3 doses).

Imatinib 2mg once a day

Sirolimus titrated to target trough level 10-15ng/ml

Everolimus 7.5mg once a day

Oral etoposide 50mg/m^2^ for 21 out of 28 days in each cycle.
